# Supplementary material for: Effect of Sodium-Glucose Co-Transporter 2 Inhibitors Combined with Metformin on Pancreatic Cancer Cell Lines
Source: Int J Mol Sci. 2024 Sep 14;25(18):9932. doi: 10.3390/ijms25189932 (PMC11432055; doi:10.3390/ijms25189932)
Supplement: Supplementary file 1 [file ijms-25-09932-s001.zip › ijms-3152231-supplementary.pdf]

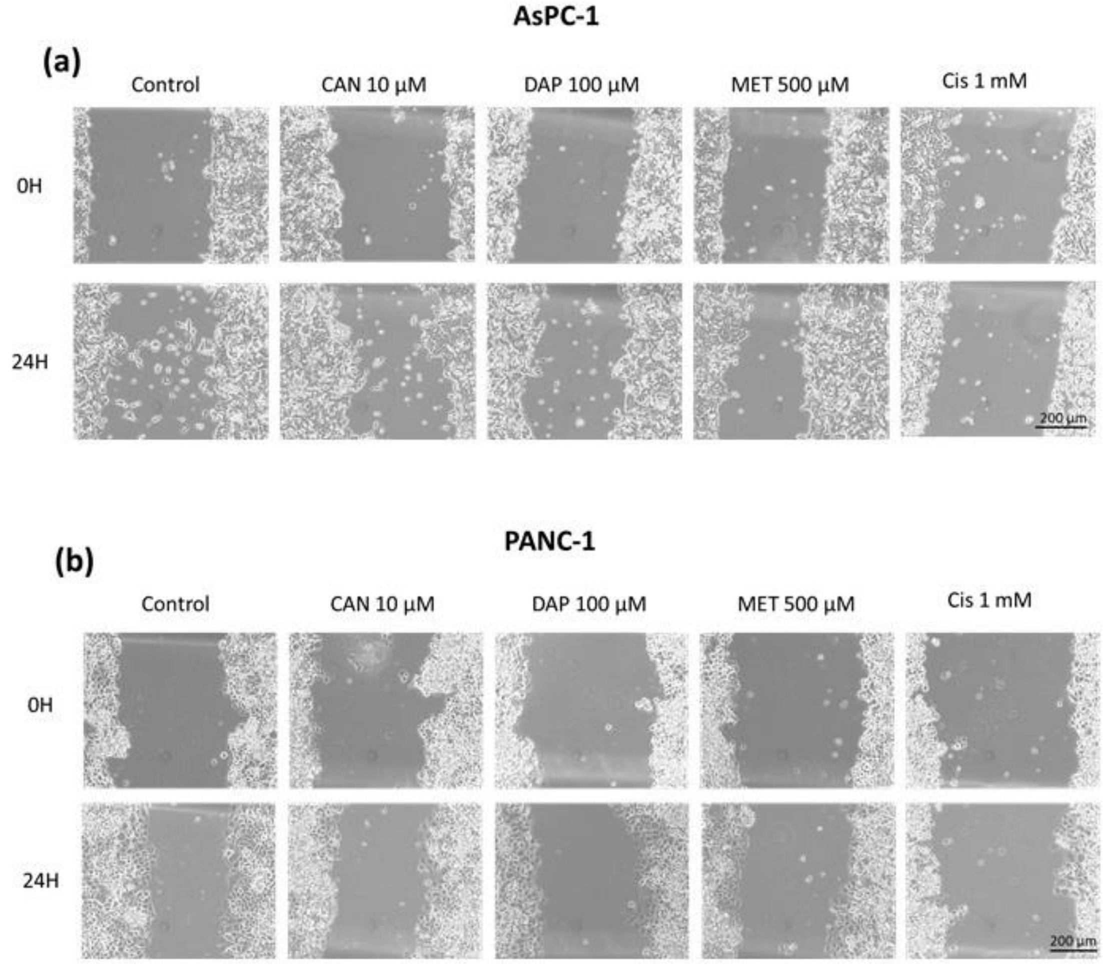

Figure S1. Effect of treatments with canagliflozin (Can), dapagliflozin (Dap), metformin (Met) and Cisplatin (Cis) on migration ability of (**a**) AsPC-1 and (**b**) PANC-1 cell lines. Shown are the representative images obtained with each condition (magnification 10 X).

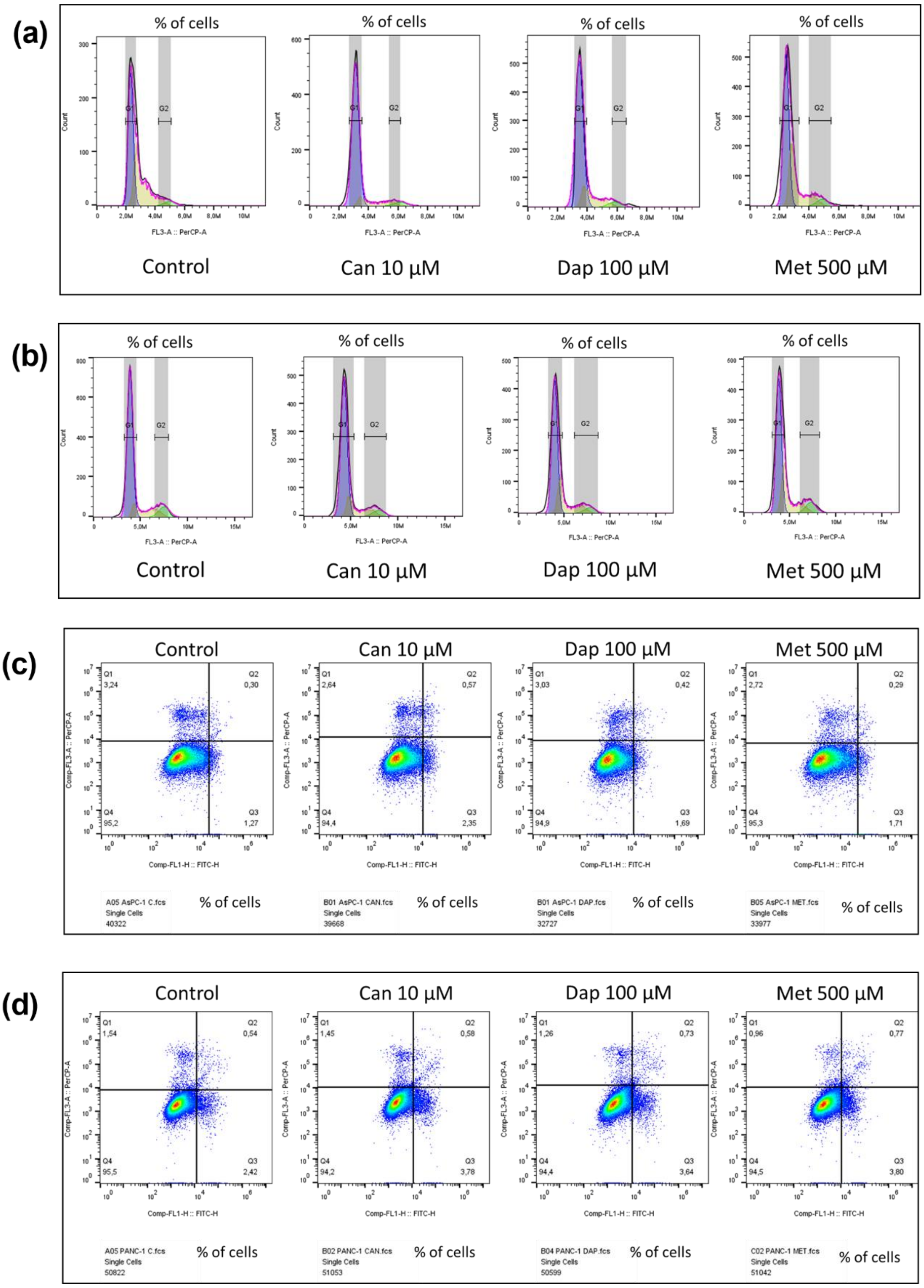

Figure S2. Representative plots of the effect of treatments with canagliflozin (Can), dapagliflozin (Dap) and metformin (Met), on cell cycle in the (**a**) AsPC-1 and (**b**) PANC-1 cell lines and on apoptosis rates in the (**c**) AsPC-1 and (**d**) PANC-1 cell lines.

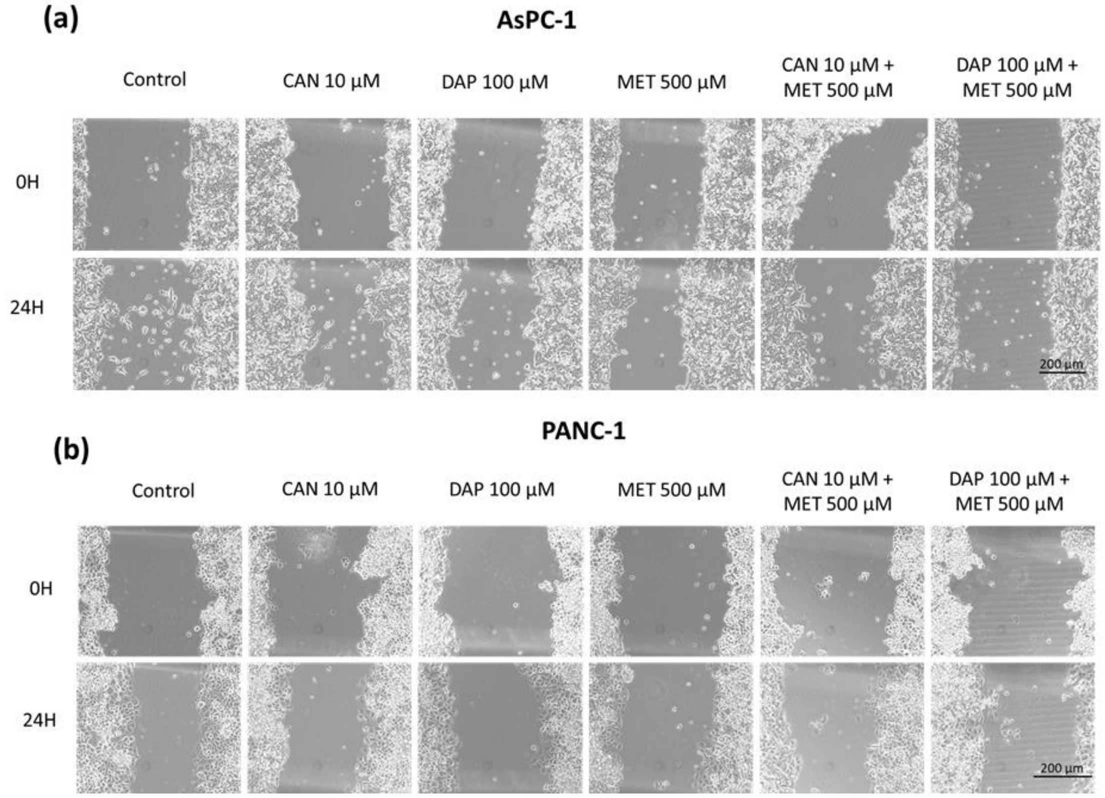

Figure S3. Effects of treatments with Canagliflozin (Can), Dapagliflozin (Dap) and Metformin (Met), separately or in combination, on the migration of the (**a**) AsPC-1 and (**b**) PANC-1 cell lines. Shown are the representative images obtained with each condition (magnification 10 X).

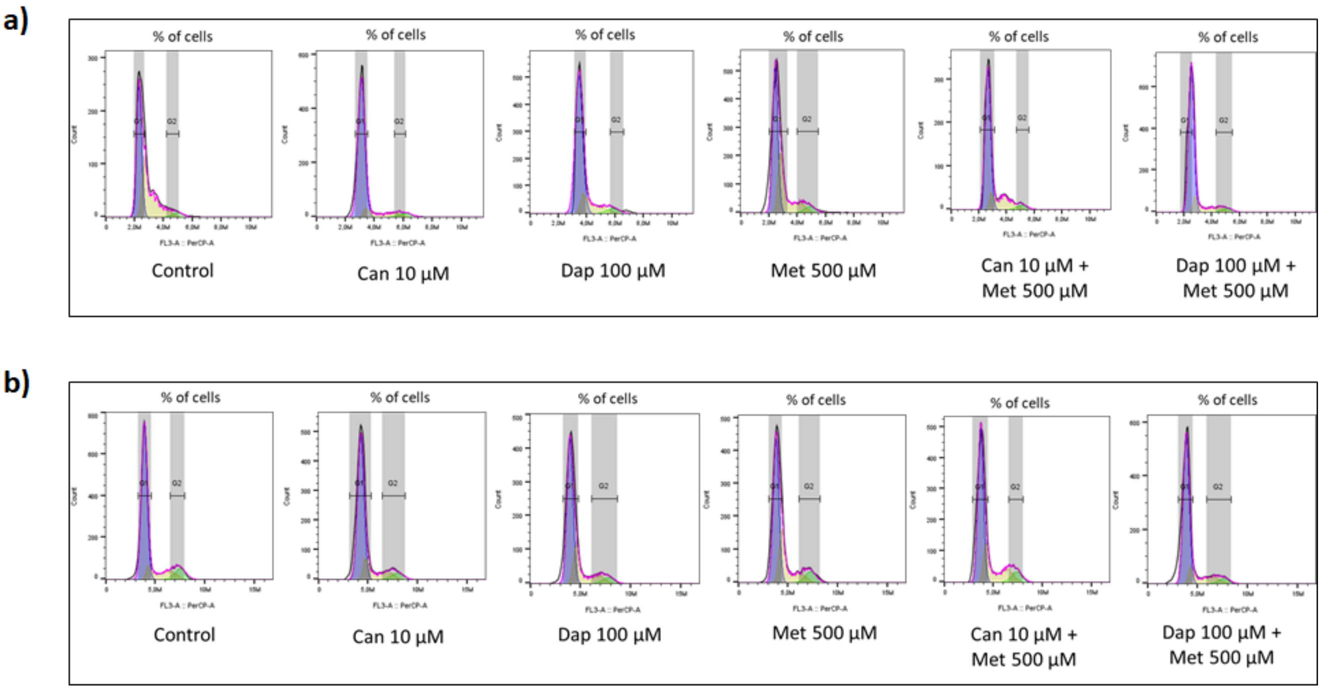
a)
% of cells
Control
Can 10 µM
Dap 100 µM
Met 500 µM
Can 10 µM + Met 500 µM
Dap 100 µM + Met 500 µM
b)
% of cells
Control
Can 10 µM
Dap 100 µM
Met 500 µM
Can 10 µM + Met 500 µM
Dap 100 µM + Met 500 µM

c)

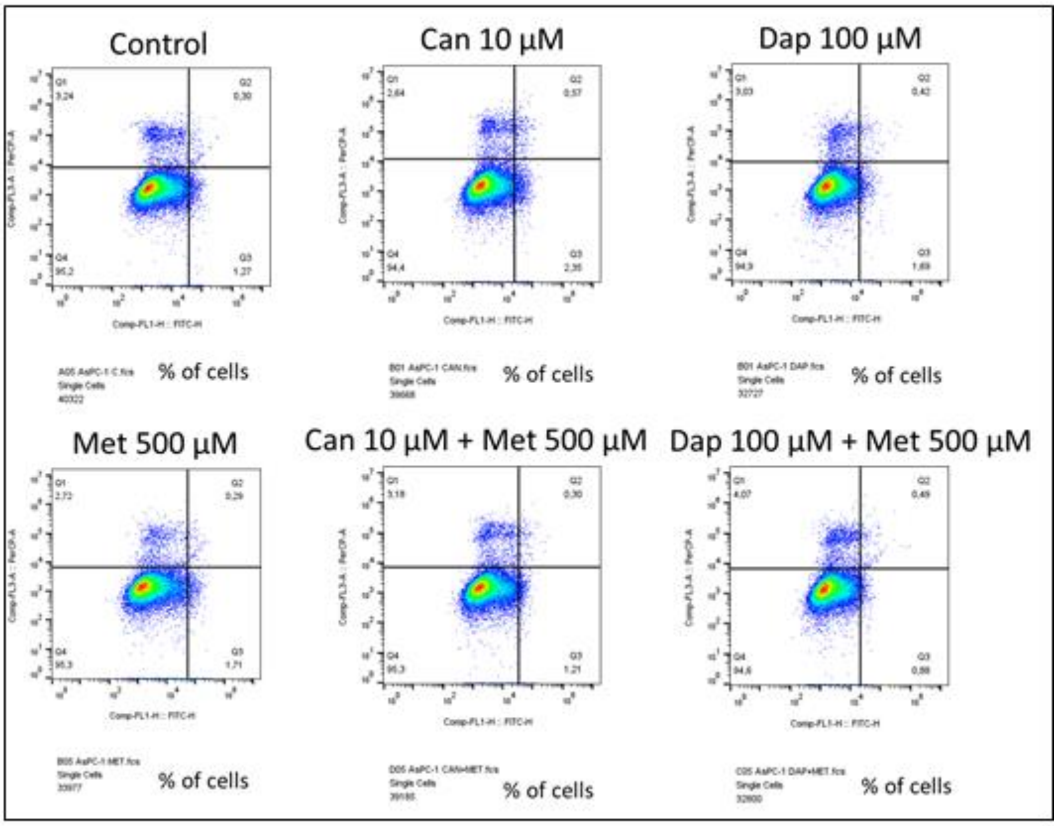

d)

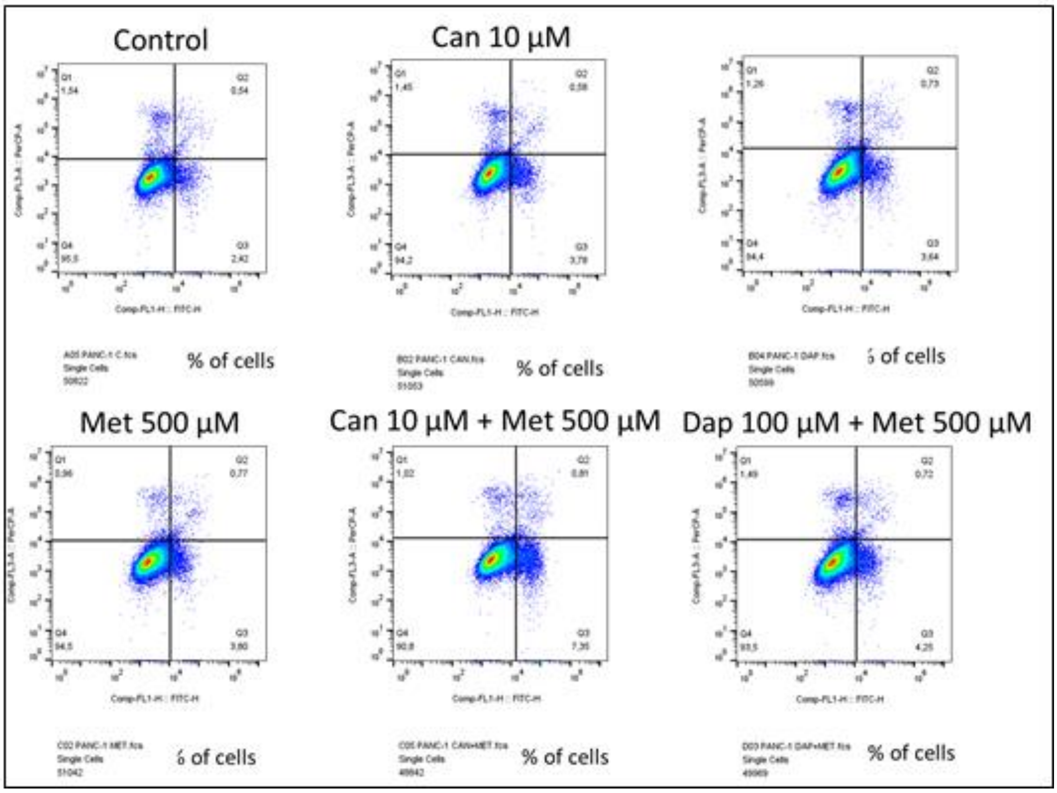

Figure S4. Representative plots of the effect of treatments with canagliflozin (Can), dapagliflozin (Dap) and metformin (Met), separately or in combination, on cell cycle in the (**a**)

AsPC-1 and (**b**) PANC-1 cell lines and on apoptosis rates in the (**c**) AsPC-1 and (**d**) PANC-1 cell lines.
